# Impact of Ceramic Material and Preparation Design on Marginal Fit of Endocrown Restorations

**DOI:** 10.3390/ma15165592

**Published:** 2022-08-15

**Authors:** Mai Soliman, Ghadeer Alzahrani, Foton Alabdualataif, Elzahraa Eldwakhly, Sahar Alsamady, Alhanoof Aldegheishem, Manal M. Abdelhafeez

**Affiliations:** 1 Department of Clinical Dental Sciences, College of Dentistry, Princess Nourah bint Abdulrahman University, P.O. Box 84428, Riyadh 11671, Saudi Arabia or; 2 General Dentistry, College of Dentistry, Princess Nourah bint Abdulrahman University, P.O. Box 84428, Riyadh 11671, Saudi Arabia; 3 Department of Conservative Dental Sciences, College of Dentistry, Qassim University, P.O. Box 6688, Buraydah 51452, Saudi Arabia; 4 Faculty of Dentistry, October University for Modern Sciences and Arts, Giza 12451, Egypt

**Keywords:** endocrown, marginal gap, celtra duo, enamic, intraradicular extension, preparation design

## Abstract

Background: The aim of this study is to investigate the impact of ceramic material and preparation design on the marginal fit of endocrown restorations. Methods: Forty endocrown restorations were CAD/CAM-fabricated for forty extracted maxillary first premolar teeth. Samples were divided into two groups (*n* = 20) according to the ceramic materials used: Celtra Duo and Vita Enamic. Each group was divided into two subgroups (*n* = 10) according to the preparation design: with no intraradicular extension and with 3 mm intraradicular extension. The marginal gap was examined using a digital microscope. Results: Celtra Duo without intraradicular extension recorded the least mean marginal gap (7.74 ± 1.55 µm), while Group Celtra Duo with 3 mm intraradicular extension recorded the highest mean marginal gap (29.54 ± 6.32 µm). Group Vita Enamic recorded a lesser marginal gap (18.03 ± 12.11 µm) than group CD (Celtra Duo) (18.64 ± 12.05 µm). There is a statistically non-significant difference between the two groups of materials (*p* = 0.873). There is a statistically significant difference between the two tested preparation designs (*p* < 0.001). Conclusion: All groups recorded a marginal gap within clinically accepted values. Material selection may influence the fitting of restorations. Intraradicular extension for endocrown restorations adversely affects the marginal fit, however, the marginal gap is still within the clinically accepted range.

## 1. Introduction

Endodontically treated teeth exhibit a high risk of biomechanical failure due to loss of tooth structure by dental trauma or caries. Therefore, their rehabilitation is considered as a clinical challenge [1]. They can be restored by post–core, crown, inlay only, and endocrown [2].

Endocrown can be defined as an adhesive restoration that has a single unit of core and crown. The advantages of endocrown restorations are that it is a conservative technique, is less time consuming, and has a reduced treatment cost. It is indicated to restore endodontically treated teeth with anatomically variated roots, calcified or curved root canals that would hinder the placement of regular post–core and with teeth that have extensive loss of coronal structure [3].

Endocrown is a Monoblock restoration that consists of a crown part and a central retainer inside the pulp chamber. It provides retention by using the pulp chamber and the cavity margins to achieve stability with adhesive cementation [4].

Cases with excessive loss of tooth structure, where there is only 1–2 mm of intact tooth structure left above the cemento-enamel junction, intraradicular extension might be needed to add further retention of the restoration. The deeper the pulp cavity that resulted from intra-coronal extension, the greater the surface area consumed for adhesive retention and the better the distribution of masticatory forces [5].

There are many factors affecting the long-term success of endocrown restorations, such as: case selection, proper coronal and intraradicular preparation, as well as internal adaptation, marginal gap and appropriate choice of bonding agent and ceramic material [2,6].

The marginal gap represents the vertical distance between the finish line and the most apical part of the crown represented [7]. Proper marginal fit is considered as a critical factor regarding the long-term success of the fixed prosthesis. Improper marginal fit will lead to exposure of the luting material to the oral environment, leading to the dissolution of the cement and the incorporation of microbacteria on the plaque, which initiate decay and subsequently the failure of the restoration [8].

The computer-aided design/computer-aided manufacturing (CAD/CAM) system has a verity of dental applications. It fabricates restorations with increased precision of marginal adaptation [1]. CAD/CAM-fabricated restorations’ fitting accuracy depends on the scanning process, milling procedure, post milling dimensional changes, and software design. Regarding the CAM process, the shape and the diameter of the milling instruments restricts the machining of the internal contour, which can affect the accuracy and the fitting of the restorations [9].

Endocrowns manufactured by CAD/CAM have been frequently used for the restoration of endodontically treated teeth. A more acceptable marginal fit, appropriate strength, and esthetics of restoration can be achieved through CAD/CAM technologies [10]. Moreover, using CAD/CAM for endocrown restoration has the advantage of minimizing chair time [11].

There are many factors influencing the fit of restorations have been described in the literature, including preparation design, parameter setting, and cement space and type. CAM machinability is a critical factor, especially for the aspect marginal region. Inadequate restoration milling leads to the inability of the restoration to seat properly and may be seated with occlusal discrepancy. If too much material is milled around the marginal area, microleakage will be the result [12].

A digital replica of the preparation is afforded by different types of scanners in STL (standard tessellation language), OBJ (Wavefront Object file), and PLY (Stanford Triangle Format) format [13]. Precise capture of critical areas with the scanners and several other parameters are essential for the accuracy of the digital impressions [14]. It is also reported by Chen et al. (2021) [15] that the use of different optical systems affects scanning accuracy.

Furthermore, Erozan and Ozan (2020) [16] found that the file type used in the restoration design stage either in a special format or STL format affects the accuracy of the impression. Another study performed by Oh et al. (2020) [17] concluded that the scanning strategy affects restoration accuracy. According to a study performed by Son and Lee (2021), [18] the margin level; equigingival or subgingival, can affect restoration accuracy. Furthermore, software design, data processing and image triangulation method affect the resolution and surface topography of the final digital impression produced [19].

Primescan is a novel intraoral scanner (Primescan, Sirona, Dental Systems Gmbh FabrikstraBe, Bensheim, Germany) which has the pioneering Smart Pixel Sensor, capable of processing more than 1,000,000 3D points/second and producing photorealistic and highly precise data. Primescan was perceived as the most accurate intraoral scanners among that investigated in in-vitro study [14]. Primescan exhibits special dynamic depth scanning technology that enables perfect image sharpness and outstanding restoration accuracy, even at a scanning depth up to 20 mm, which provides a supreme advantage for deeper-lying indications. In addition, the wider field of view visualizes larger areas with less sweeps and immediate accuracy. It can also consolidate more than 50,000 images/second that results in fast processing of the exact data that the software needs.

Current CAM systems that gather up four or five milling were the focus of studies which have shown that the five-axis milling machines are defined by special precision [20,21]. Again, another study has shown that the five-axis CAM component presented superior accuracy than those with four axes. Furthermore, close attention is given to the diameter of the cutter: the smaller the diameter is, the more accurate the prosthesis [22].

Recently, a wide range of ceramic materials have been developed exhibiting improved mechanical and esthetic properties to advance the clinical performance and to satisfy the patient esthetic demand [23]. A wide variety of materials are used for CAD/CAM manufacturing of endocrown restoration, such as lithium disilicate ceramic, polymer infiltrated ceramic, zirconia-reinforced lithium silicate ceramic and resin nanoceramic [24,25,26].

Different material characteristics affect their machinability with CAD/CAM milling machines. Celtra Duo is a type of a high-strength CAD/CAM ceramic that was introduced in 2012 as zirconia-reinforced lithium silicate (ZLS). It has a unique composition of Zirconium dioxide (10%) that increases the material strength, yet allows it to be machined. It is recommended for anterior and posterior single-unit crown restorations as well as inlays, onlays, and veneers [27].

ZLS-ceramics consist of two components: small crystalline lithium metasilicate with lithium disilicate crystals with average size: 0.5–0.7 μm. The second is the glassy matrix with 10% zirconium oxide. This microcrystalline configuration grants for an optimal flexural strength, moreover, it still has a high proportion of glassy matrix, which offers good polishing and optical properties [28,29].

Consequently, the final crystallized ZLS modifications present an ideal integration of reduced manufacture times and high stability. These variations promote the chairside fabrication of posterior all-ceramic prosthesis [30].

CAD/CAM composites, which have been recently released to the market, have been recommended for manufacturing monolithic cemented restorations. CAD/CAM composites are divided into two types, namely, hybrid and polymer-infiltrated ceramic network (PICN) materials [31]. A PICN material consists of 75% glass ceramic and dimethacrylate monomer polymerized at high pressure and temperature by volume, thereby combining the properties of ceramic and polymer [32].

A PICN material contains a hybrid network structure with two interpenetrating phases; an 86% inorganic phase, including feldspathic ceramic as the dominant phase, and a 14% organic phase composed of dimethacrylates. The dominant ceramic network provides resistance to deformation and wear, but it is susceptible to fracture [33].

PICN, introduced in 2013 as Vita Enamic, is a resilient CAD/CAM ceramic that gives an accurate and fast milling as well as competent finishing and polishing [34]. Vita Enamic is specifically suited for minimally invasive restorations, crown restorations exposed to high masticatory forces (molar area) and cosmetic veneer restorations [35], as well as its classic indication for single tooth restorations such as inlays, onlays, veneers and crowns.

Vita Enamic as a new restorative material exhibits one main advantage which is the reasonable brittleness index that makes the material a suitable to be a CAD/CAM candidate [20]. One step manipulation material also ensures a high degree of dimensional accuracy of the final restorations. Moreover, it has comparable creep response as enamel and low hardness that grant lower contact stresses and better stress redistribution [36].

Resin-based CAD/CAM blocks show more margin stability when compared to ceramic blocks. To ensure well-fitting restorations, ceramic-based materials may require certain material thicknesses, as they are more brittle and thus more susceptible to fracture. The fitting accuracy of restorations has been shown to influence the clinical long-term success of restorations [12].

Accordingly, as there are limited data available about the marginal integrity of endocrown restorations and insufficient information about the effect of preparation designs and materials on the fit of endocrown restorations [37], further investigations are always required to explore the biomechanical behavior of recent materials when used for endocrown restorations before being tried clinically.

Therefore, this in vitro study was conducted to investigate the impact of CAD/CAM restorative ceramic materials; Celtra Duo (zirconia-reinforced lithium silicate) and Vita Enamic (polymer-infiltrated ceramic network), and preparation design; with 3 mm intraradicular extension, and without intraradicular extension, on the marginal gap of endocrown restorations.

The tested hypothesis of the study is that there will be a non-significant difference in the marginal fit of the two tested ceramic materials used for the fabrication of endocrown. While there will be a significant difference in the marginal fit of the two tested preparation designs, speculating that the preparation design without intraradicular preparation will record a better marginal fit.

## 2. Materials and Methods

### 2.1. Natural Teeth Collection

Forty extracted sound human maxillary first premolars indicated for extraction for orthodontic treatment were collected and were cleaned (SUPRASSONTM P5 Booster ultra-sonic scaler, France). The teeth were then stored at room temperature in 0.1% Thymol solution (Caelo, Hilden, Germany) until required for experimentation. The inclusion criteria for the selected teeth included completely formed apices, absence of carious lesion, fracture lines and cracks and similar bucco-lingual (BL) and mesio-distal (MD) dimensions as determined with a digital caliper. The teeth were visually inspected under magnification of 2.5× magnifying loupes.

### 2.2. Sample Grouping

Teeth were divided randomly into two groups according to the ceramic materials used: Group CD; Celtra Duo (Dentsply, Sirona, Dental Systems Gmbh FabrikstraBe, Bensheim, Germany) and Group VE; Vita Enamic (Vita, Vita Zahnfabrik, Bad Säckingen, Germany) (Table 1).

Each group was further sub-divided into two subgroups (*n* = 10) according to the endocrown preparation design: Subgroup W; with 3 mm intraradicular extension, and subgroup WO; without intraradicular extension.

- The two tested groups are:
  - CD; Celtra Duo.
  - VE; Vita Enamic.
- The two tested sub-groups are:
  - CD-W; Celtra Duo with 3 mm intraradicular extension.
  - CD-WO; Celtra Duo without intraradicular extension.
  - VE-W; Vita Enamic with 3 mm intraradicular extension.
  - VE-WO; Vita Enamic without intraradicular extension.

### 2.3. Coronal Preparation

Teeth were decoronated perpendicularly to their long axis at 3 mm occlusal to the most occlusal point of the proximal CEJ, and pulp chamber was prepared to have smooth divergent walls using diamond burs under copious water coolant.

### 2.4. Endodontic Treatment of All Teeth

The working length (WL) was established, and radiographs were taken for WL confirmation. Root canals were cleaned and shaped to the WL using X-Smart rotary contra-angle motor (Dentsply, Sirona, Canada) with Protaper Next (PTN) rotary filing system (Dentsply; Maillefer, Ballaigues, Switzerland) at a speed of 300 rpm and under a torque of 2.5 Ncm. Teeth were consequently irrigated throughout the steps of preparation using 6.15% NaOCl (The Clorox Co., Oakland, CA, USA), with a final flush of 17% EDTA (PULPDEN Corporation, Oakland, Watertown, MA, USA). Finally, the prepared teeth were obturated with gutta-percha points size X2 of the same PTN system and according to manufacturer instructions using Bioceramic sealer (Brasseler USA, Savannah, GA, USA).

### 2.5. Intraradicular Preparation

**Figure 1:**
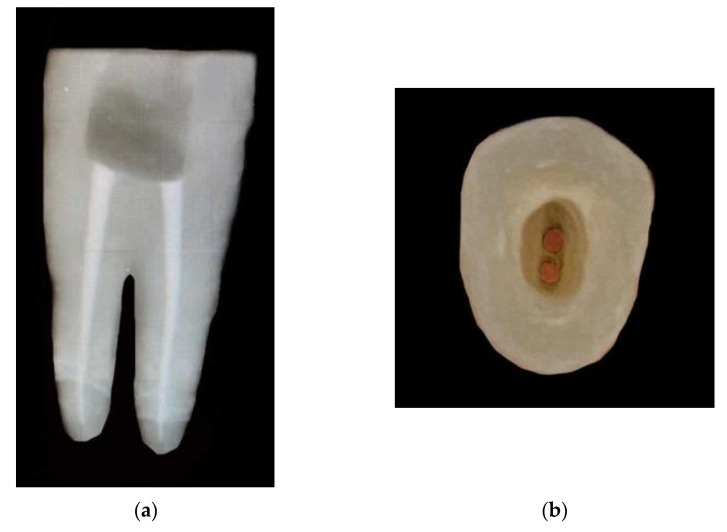
(**a**) Radiographic proximal view of Endocrown preparation without intraradicular extension. (**b**) Occlusal view of Endocrown preparation without intraradicular extension.

**Figure 2:**
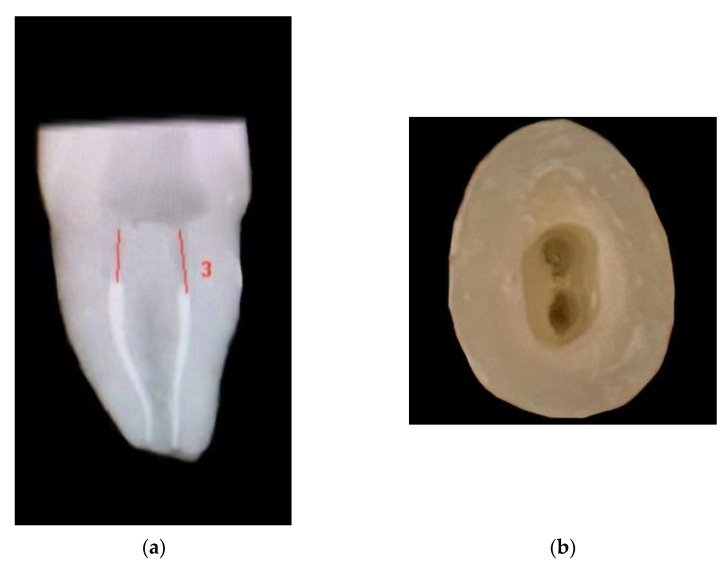
(**a**) Radiographic proximal view of Endocrown preparation with 3 mm intraradicular extension. (**b**) Occlusal view of Endocrown preparation wit 3 mm intraradicular extension.

For groups CD-WO and VE-WO; removal of 3 mm of gutta-percha from both the buccal and the palatal canal was performed using Lexicon Gates Glidden Drills (Dentsply, Sirona, PA, USA) (Figure 1a,b and Figure 2a,b).

### 2.6. CAD/CAM Fabrication of Endocrown Restoration

**Figure 3:**
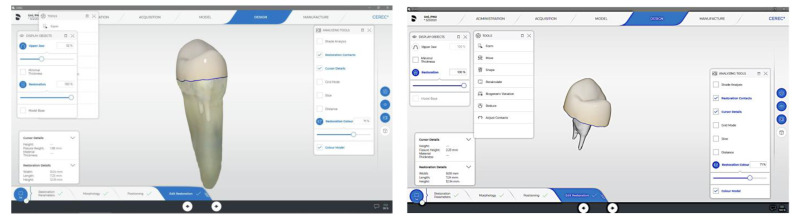
Primescan generated design of endocrown with 3 mm intraradicular extension.

**Figure 4:**
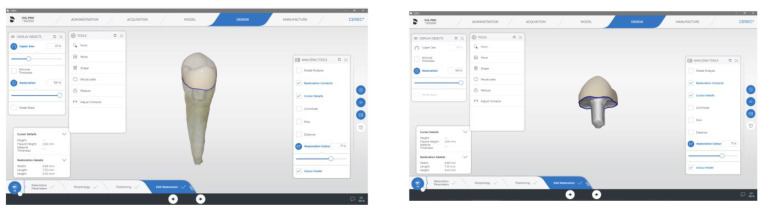
Primescan generated design of endocrown without intraradicular extension.

**Figure 5:**
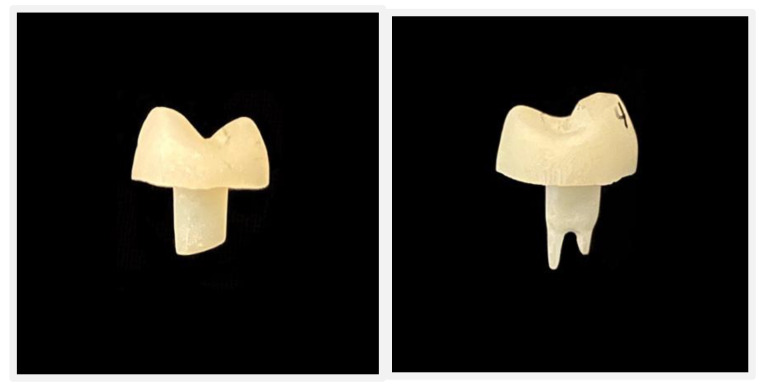
CAD/CAM-fabricated endocrown restoration.

Digital impressions were performed via digital scanner (Primescan, Sirona, Dental Systems Gmbh FabrikstraBe, Bensheim, Germany). Endocrowns were designed using a software package (CEREC 3D, version 5.2, Sirona Dental Systems GmbH, Bensheim, Germany). Endocrown restorations were milled using the CEREC MC XL Milling Machine (Sirona, Dental Systems Gmbh FabrikstraBe, Bensheim, Germany) (Figure 3, Figure 4 and Figure 5).

### 2.7. Marginal Gap Testing

**Figure 6:**
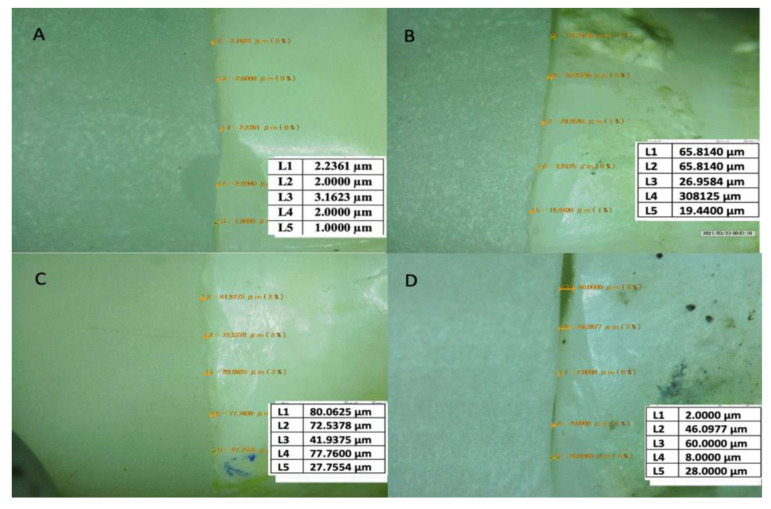
Marginal gap measuring using a digital microscope. (**A**) Celtra Duo without intraradicular extension. (**B**) Celtra Duo with 3 mm intraradicular extension. (**C**) Vita Enamic without intraradicular extension. (**D**) Vita Enamic with 3 mm intraradicular extension.

The marginal gap was examined using a digital microscope (KH-7700 Hirox Company, Hackensack, NJ, USA) under ×40 magnification. Marginal gap was measured at eight predetermined points; midbuccal, mesiobuccal, distobuccal, midlingual, mesiolingual, distolingual, mid-distal and mid-mesial. Endocrown restoration was stabilized to the prepared tooth using a custom-made holder with a special pin to lock the specimen in place. Measurements were recorded and tabulated for statistical analysis (Figure 6).

### 2.8. Statistical Analysis

Statistical analysis was performed using IBM SPSS software package version 20.0 (IBM Corp., Armonk, NY, USA). The Shapiro–Wilk test was used to prove the normality of distribution. For continuous data the distributed data were expressed as range (minimum and maximum), mean and standard deviation. Two-way (ANOVA) was performed to identify significant differences in marginal fit according to the two tested materials, as well as the two tested preparation designs. The significant difference in the marginal fit according to interaction between both tested materials and designs was also evaluated. Student t-test at a significance level of *p* ≤ 0.05 was then used to compare between the two groups and the two subgroups.

## 3. Results

**Figure 7:**
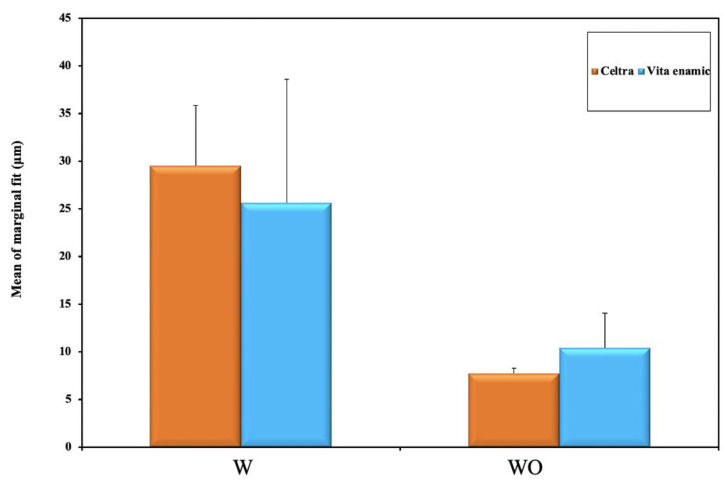
Graphical representation of mean marginal fit (μm) according to the two studied materials and preparation design.

Group C-WO: Celtra Duo without intraradicular extension, recorded the least mean marginal gap (7.74 ± 1.55 µm), followed by group E-WO: Enamic without intraradicular extension, (10.42 ± 3.63 µm), and group E-W: Enamic with 3 mm intraradicular extension, (25.63 ± 12.96 µm). Group C-W: Celtra Duo with 3 mm intraradicular extension, recorded the highest mean marginal gap (29.54 ± 6.32 µm) (Table 2, Figure 7).

Two-way ANOVA showed that the interaction between the material and the preparation design was not significant (*p* = 0.172), nor the effect of the material (*p* = 0.796). Only the effect of designs showed any significant effect (*p* < 0.001) with the tested types of materials (Table 3).

**Figure 8:**
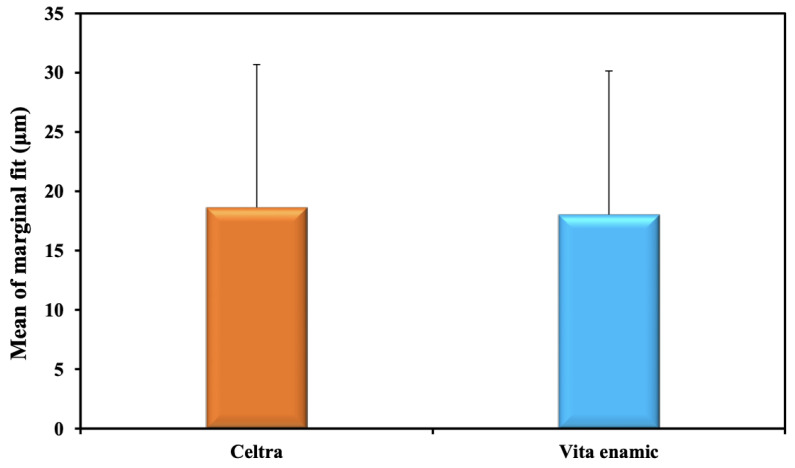
Comparison between marginal fit (μm) of the two studied materials.

Regarding the effect of material on the marginal gap regardless of the preparation design, group E; Enamic, recorded less marginal gap (18.03 ± 12.11 µm) when compared to group C; Celtra Duo, (18.64 ± 12.05 µm). However, there is a statistically non-significant difference between the two material groups (*p* = 0.873) (Table 4) (Figure 8).

**Figure 9:**
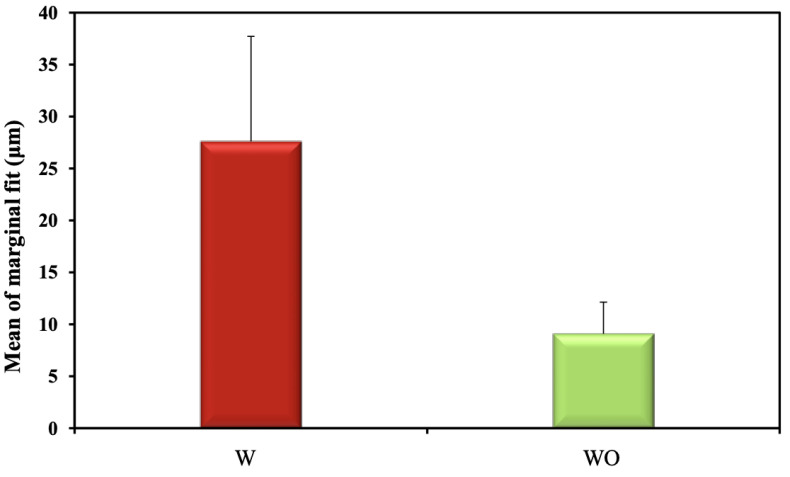
Comparison between marginal fit (μm) of two studied preparation designs.

Regarding the effect of preparation design on the marginal gap regardless of the material used, a statistically significant difference was observed between the two tested preparation designs (*p* < 0.001), as group WO; without intraradicular extension, recorded less marginal gap (9.08 ± 3.04 µm) when compared to group W; with 3 mm intraradicular extension, (27.59 ± 10.12 µm) (Table 5, Figure 9).

## 4. Discussion

The hypothesis of this study is accepted. The results confirmed a non-significant difference in the marginal fit of the two tested ceramic materials used for endocrown fabrication. Whereas a significant difference in the marginal fit was recorded for the two tested preparation designs. The preparation design without intraradicular extension recorded a better marginal fit when compared to the preparation design with intraradicular extension.

Endocrowns are restorations fabricated to restore endodontically treated teeth without the post design by the means of the macro-retentive support from the pulp chamber walls as well as the micromechanical retention achieved by adhesive cementation [1].

Computer-aided design/computer-aided manufacture (CAD/CAM) system has a huge advancement in the variety of materials used for dental treatments. These materials have similar mechanical properties to natural tooth structure. The ceramic materials used are CAD/CAM blocks: CELTRA DUO (zirconia-reinforced lithium silicate) and Vita Enamic (polymer-infiltrated ceramic) [38]. CAD/CAM technology has a continues development as it provides a marginal fit better than that of the restorations that are lab-fabricated [39].

In the current study, human teeth have been used for resembling the clinical situation regarding the presence of enamel and dentin architecture and contours of pulp chambers and root canals. However, they might have caused some degree of variability in the results because of the difficulty in standardization [40].

In the current study, all the CAD/CAM procedures were standardized for all the tested groups to obtain the best results for each type of tested materials. In addition, milling procedures for brittle surfaces such as ceramics were performed without much pressure applied on the margins [22]. Milling instruments were also changed after every ten restorations, as the wear of milling machine tools is affected by material composition. The stronger the CAD/CAM material, the higher the wear rate of milling instruments. Moreover, the longer the milling tools are used, the worse the fit outcome and the roughness of the final restorations [12].

In 2017, a study was performed by Manmohan et al. in which they tested the marginal fit of zirconia copings milled with 4-axis and 5-axis milling machines, and the results showed that the accuracy of the 5-axis milling machine is higher than the 4-axis milling machine [41].

In the present study, digital microscopy, a non-invasive evaluation method, was used to measure the marginal fit of the endocrown restorations. All measurements were performed by the same operator for the purpose of standardization [5]. The selection of digital microscopy is due to its noninvasive nature, as it does not require sectioning or replications before measuring the gap [42,43].

The vertical marginal gap measurement was selected in the current study as it is the most frequently used to quantify the fit of a restoration [44]. The butt margin design used in this study offers a simple marginal configuration without thin margins which contributed to the proper seating of the endocrown restorations which affects the vertical marginal gaps [45].

Results of the present study revealed that Group C-WO: Celtra Duo without intraradicular extension, recorded the least mean marginal gap (7.74 ± 1.55 µm), followed by group E-WO: Enamic without intraradicular extension, (10.42 ± 3.63 µm), and group E-W: Enamic with 3 mm intraradicular extension, (25.63 ± 12.96 µm). Group C-W: Celtra Duo with 3 mm intraradicular extension, recorded the highest mean marginal gap (29.54 ± 6.32 µm).

Results of the current study revealed that there was a non-significant difference of marginal fit between the two studied ceramic materials (*p* = 0.873). This result coincides with a study conducted by Taha D et al. in 2018, which discussed the marginal fit of endocrown restorations using materials such as zirconia-reinforced lithium silicate ceramics (Celtra Duo) and polymer-infiltrated ceramics (Vita Enamic), and they concluded that these materials showed a marginal gap that is clinically acceptable [25].

Comparing the results with the study by Mahya Hasanzade (2019), neither type of restoration nor material affected the marginal gap. In their study they tested zirconia-reinforced lithium silicate (Suprinity), polymer-infiltrated ceramic network (Enamic) and lithium disilicate (IPS e-max CAD) [42].

Moreover, El Ghoul et al. (2020) discussed the effect of different CAD/CAM materials on endocrown marginal fit. They tested hybrid nanoceramic (Cerasmart), hybrid nanoceramic (Cerasmart), lithium disilicate glass-ceramic (IPS e.max CAD) and zirconia-reinforced lithium silicate glass-ceramic (Vita Suprinity) and concluded that all the materials provided an acceptable result [46].

Despite the fact that there is a statistically non-significant difference between the two material groups on the marginal gap regardless of the preparation design, group E: Enamic, recorded less marginal gap when compared to group C: Celtra Duo). This could be explained by the fact that the hybrid ceramic is composed of a structure-sintered ceramic matrix (86 wt%) and a reinforcing polymer network (14 wt%), which are fully integrated with one another and provide considerable benefits for the user, including its lower brittle fracture tendency compared to pure ceramics and excellent CAD/CAM processing application. Vita Enamic also expresses high edge quality as it could be milled in thinner sections without being deformed, providing extremely precise milling results [47,48]. Consequently, results found in the current study are in accordance with the material characteristics described in the literature regarding resin-based composite material restorations having a higher marginal stability than ceramic materials [48].

Regarding the clinical acceptable marginal gap, there is some controversy in the literature. However, many authors concluded that marginal gaps below 120 μm are considered clinically acceptable [5]. The results of the current study revealed that both tested materials and preparation designs recorded marginal gap values that are within clinically accepted values.

Sağlam et al. (2020) reported different clinical marginal gaps between CAD/CAM-fabricated endocrowns from Feldspathic ceramic block and Polymer-infiltrated ceramic network block. CAD/CAM-fabricated endocrowns are an option for the restoration of endodontically treated teeth but their use on premolars must be evaluated carefully because mesio-distal furcation and right-angled occlusal anatomy between the cusps make these teeth susceptible to vertical fractures in the mesio-distal direction [49].

Results of the present study revealed that there was significant difference of the marginal fit between two studied preparation designs (*p* values < 0.001). As the preparation design without intraradicular extension recorded better marginal fit. This finding is similar to a study by Gaintantzopoulou MD. (2016), who stated that an intraradicular extension of 2 mm inversely affected the marginal fit of the restorations [5].

Elalem IA et al. (2019) tested the marginal fit of E-Max endocrown restorations with different preparation designs as group 1 had a preparation with butt joint and group 2 with a circumferential preparation and a chamfer finish line. The marginal gaps of both groups were within the clinically acceptable range, but group 1 with butt joint preparation was statistically significantly higher than group 2 with a circumferential preparation and a chamfer finish line. Meanwhile, there was no significant difference regarding the internal fit of both groups [6].

Moreover, Ali M. et al. (2021) concluded that increasing the cavity depth preparation design will inversely affect the marginal fit, as 2 mm cavity depth showed a significantly lower marginal gap than a 4 mm cavity depth [50].

A study conducted by Gurpiner B. et al. in 2021 discussed the accuracy of endocrown scans in different intrapulpal depth designs, and it concluded that CEREC Primescan was found to have the highest precision and trueness compared to other intraoral scanners [51].

Limitations of this study include that it is an in vitro study, which may differ from a clinical study, where the scanning procedure would be less precise due to limitations such as the presence of saliva and access limitation of the scanner in the oral cavity. Therefore, further in vivo clinical studies are recommended [52]. Furthermore, the anatomical variations among the forty natural teeth used in this study might result in discrepancy. In addition, measurements of the specimens without cementation can be listed as one of the study’s limitations.

Overall, an evidence-based approach should be presented concerning the clinical use of endocrown restorations to be assembled using intraoral scanners and the performance of intraoral scanners in future clinical studies.

## 5. Conclusions

Within the limitations of this in vitro study, the following conclusions may be drawn:

1. Both tested materials and preparation designs recorded marginal gap values that are within clinically accepted values.
2. CAD/CAM material selection may influence the fitting accuracy of CAD/CAM-fabricated restorations, as resin-based hybrid ceramic material recorded least marginal gab when compared to zirconia-reinforced lithium silicate.
3. Intraradicular extension for endocrown restorations adversely affects marginal fit, however, the marginal gap is still within a clinically accepted range.

## Figures and Tables

**Table 1:** The two tested materials’ composition (Celta Duo and Vita Enamic).

Material	Manufacturer	Ceramic Type	Chemical Composition
**Celtra Duo**	Dentsply, United States	zirconia-reinforced lithium silicate ceramic	SiO_2_, P_2_O_5_, Al_2_O_3_, Li_2_O, ZnO, 10% ZrO_2_
**Vita ENAMIC**	VITA-Zahnfabrik, Germany	polymer infiltrated ceramic	Polymer-infiltrated-feldspatic ceramic-network material (UDMA, TEGDMA) with 86 wt% ceramic (SiO_2_, Al_2_O_3_, Na_2_O, K_2_O, B_2_O_3_, CaO, TiO_2_, coloring oxides)

**Table 2:** Measurements of marginal fit (μm) according to the two studied materials and preparation designs.

	Materials		Total Designs
Designs	Group C; Celtra Duo	Group E, Enamic	
W, with 3 mm extension	(*n* = 10)	(*n* = 10)	(*n* = 20)
Min.–Max.	18.97–36.16	7.42–43.93	7.42–43.93
Mean ± SD.	29.54 ± 6.32	25.63 ± 12.96	27.59 ± 10.12
WO; without extension	(*n* = 10)	(*n* = 10)	(*n* = 20)
Min.–Max.	5.69–9.84	7.66–17.20	5.69–17.20
Mean ± SD.	7.74 ± 1.55	10.42 ± 3.63	9.08 ± 3.04
Total Materials	(*n* = 20)	(*n* = 20)	(*n* = 40)
Min.–Max.	5.69–36.16	7.42–43.93	5.69–43.93
Mean ± SD.	18.64 ± 12.05	18.03 ± 12.11	18.33 ± 11.93

SD: Standard deviation.

**Table 3:** Two-way ANOVA used to compare marginal fit (μm) between the studied materials and preparation designs.

Source	Type III Sum of Squares	DF	Mean Square	F	*p*
Corrected Model	3537.1	3	1179.0	21.100	<0.001 *
Materials	3.8	1	3.8	0.068	0.796
Designs	3424.8	1	3424.8	61.291	<0.001 *
Materials * Designs	108.5	1	108.5	1.942	0.172
Error	2011.6	36	55.9		
Corrected Total	5548.7	39			

F, *p*: f and *p* values for the model. DF: Degree of freedom. *: Statistically significant at *p* ≤ 0.05.

**Table 4:** Comparison between marginal fit (μm) of the two studied materials.

	Materials		*p*
	Celtra (*n* = 20)	Vita Enamic (*n* = 20)	
**Marginal fit**			
Mean ± SD.	18.64 ± 12.05	18.03 ± 12.11	**0.873**

*p*: *p* value for Student *t*-test for comparing between the different materials. Statistically significant at *p* ≤ 0.05.

**Table 5:** Comparison between marginal fit (μm) of two studied preparation designs.

	Designs		*p*
	W (*n* = 20)	WO (*n* = 20)	
**Marginal fit**			
Mean ± SD.	27.59 ± 10.12	9.08 ± 3.04	< 0.001 *

*p*: *p* value for Student *t*-test for comparing between the different designs. *: Statistically significant at *p* ≤ 0.05.

## Data Availability

Not applicable.
